# Bibliometric study of soluble guanylate cyclase stimulators in cardiovascular research based on web of science from 1992 to 2021

**DOI:** 10.3389/fphar.2022.963255

**Published:** 2022-08-23

**Authors:** Xiao-Yan Jia, Yong-Ming Liu, Yong-Fei Wang, Jin-Yang An, Ke-Ling Peng, Hua Wang

**Affiliations:** ^1^ The First Clinical Medical College of Lanzhou University, Lanzhou, Gansu, China; ^2^ Department of Geriatric Cardiology, The First Hospital of Lanzhou University, Lanzhou, Gansu, China

**Keywords:** cardiovascular research, CiteSpace, bibliometrics, heart failure, soluble guanylate cyclase stimulators, web of science, VOSviewer

## Abstract

**Background:** Several studies have shown that soluble guanylate cyclase (sGC) stimulators have cardiovascular (CV) benefits. However, few bibliometric analyses have examined this field systematically. Our study aimed to examine the publications to determine the trends and hotspots in CV research on sGC stimulators.

**Methods:** Publications on sGC stimulators in CV research were retrieved from the Web of Science Core Collection. VOSviewer and CiteSpace visualization software were used to analyze publication trends, countries (regions) and institutions, journals and cited journals, authors and cited references, as well as keywords.

**Results:** A total of 1,212 literatures were obtained. From its first appearance in 1992–2021 (based on WOSCC record), the overall volume of publications has shown a gradual increasing trend. Nearly one-third were authored by American scholars, and most were published in Circulation, Circulation Research, and Proceedings of the National Academy of Sciences of the United States of America. Bayer Agency in Germany was the leading driving force, and has a high academic reputation in this field. Stasch JP has published the most related articles and been cited most frequently. Half of the top 10 co-cited references were published in the leading highly co-cited journal Circulation and New England Journal of Medicine. “NO,” “allosteric regulation” and “free radicals” were the focus of previous research, “chronic thromboembolic pulmonary hypertension,” “pulmonary hypertension” and “heart failure” were the main research hotspots. The key words “chronic thromboembolic pulmonary hypertension,” “Pulmonary hypertension,” “preserved ejection fraction” and “heart failure” appeared most recently as research frontiers.

**Conclusion:** The research in the CV field of sGC stimulators was relatively comprehensive, and there was a close relationship among countries, research institutions and authors, but it is still in the exploratory stage in the treatment of CV disease. At present, most studies focus on the results of clinical trials. sGC stimulators in the treatment of heart failure, especially heart failure with preserved ejection fraction, may be the hotpots and Frontier at present and in the future, and should be closely monitored.

## 1 Introduction

The nitric oxide (NO)/soluble guanylate cyclase (sGC)/cyclic guanosine monophosphate (cGMP) pathway is one of the major signaling pathways involved in cardiovascular (CV) diseases, including vasoconstriction, tissue fibrosis, oxidative stress, and inflammation. sGC is a key signal transduction enzyme in the CV system. The pathogenesis of various diseases, particularly of the CV system, such as hypertension, pulmonary hypertension, heart failure, chronic kidney disease, and erectile dysfunction, is related to the dysfunction of the sGC stimulator in the NO/sGC/cGMP signaling pathway. The discovery of sGC stimulators can be said to be a milestone in the field of NO/sGC/cGMP pharmacology. Targeted drug sGC stimulators based on this signaling pathway have been developed for CV system diseases. NO production is reduced owing to endothelial dysfunction in the pathological changes of heart failure, coronary artery disease, pulmonary hypertension, and hypertension; therefore, heme-dependent sGC activation and cGMP production are reduced. In this case, there is only one way to stabilize cGMP levels: sGC stimulators directly stimulating sGC. Moreover, sGC stimulators directly bind to reduced heme-containing enzymes, resulting in an increase in cGMP production. (Schmidt et al., 2009) Therefore, sGC stimulants have potential significance in the targeted treatment of CV diseases.

Riociguat was the first sGC stimulator to successfully transition from animal experiments to controlled clinical studies in patients; and sGC stimulators, including riociguat, have shown beneficial effects in animal models of the cardiovascular system and a variety of other diseases. In preclinical models of pulmonary atrial hypertension (PAH), an sGC stimulator reduced pulmonary hypertension, right ventricular hypertrophy, and pulmonary vascular remodeling in chronic hypoxic models of PAH, and reversed hemodynamic and structural changes in rats with severe pulmonary hypertension. (Dumitrascu et al., 2006) Similarly, in a rat model of hypoxic PAH, riociguat reduced right ventricular hypertrophy, increased cardiac output, and decreased total pulmonary resistance. (Lang et al., 2012) Later, guinea pig and dog models were also added, which consistently demonstrated that riociguat treatment can significantly reduce pulmonary arterial pressure in models of PAH. These preclinical studies predict that sGC stimulators can reduce pulmonary vascular resistance (PVR) and NT-proBNP as well as improve exercise tolerance in patients with PAH, providing the basis for regulatory approval of riociguat in patients with PAH. Of course, phosphodiesterase (PDE) inhibitors are also approved for PAH, such as sildenafil and tadalafil, which can also increase cGMP levels by reducing the degradation of cGMP. However, because PDEs have multiple families that can degrade cGMP, once PDE5 is blocked, other PDEs that degrade cGMP may compensate. Furthermore, in the presence of very low endogenous NO production, which results in low endogenous cGMP levels, the efficacy of PDE5 inhibitors may be greatly limited. In contrast, sGC stimulators are independent of NO, and thus compensate for this deficiency. Subsequently, riociguat was tested in two pivotal phase 3 clinical trials, the PATENT-1 and CHEST-1 studies, in patients with PAH and chronic thromboembolic pulmonary hypertension (CTEPH), respectively (Ghofrani et al., 2013a; Ghofrani et al., 2013b). Based on clinical trial results, the US FDA approved riociguat for the treatment of CTEPH and PAH. So, it can be said that it overcomes the limitations of PAH drug treatment options.

As previously mentioned, the NO/sGC/cGMP pathway is a key regulator of the cardiovascular system. Therefore, sGC stimulators may also be an alternative to cardiopulmonary circulation and have potential for other indications. Ventricular diastolic impairment and decreased cardiac output were found in sGC-α-1 knockout mice, while an sGC stimulator could prevent cardiomyocyte hypertrophy, indicating that it had a direct effect on heart failure. (Irvine et al., 2012) In a diabetic cardiomyopathy model, sGC agonists also reduced cardiac hypertrophy and improved cardiac systolic and diastolic functions. (Mátyás et al., 2015) Furthermore, sGC stimulators have shown potent, dose-dependent hypotensive effects in salt-sensitive models of hypertension. (Tobin et al., 2018) Studies have also shown that in a myocardial infarction model, sGC stimulators can reduce infarct size and preserve ejection fraction. (Methner et al., 2013; Bice et al., 2014) NO/sGC/cGMP signaling plays an important role in regulating inflammation, which is also considered a common underlying pathophysiological mechanism in heart failure with preserved ejection fraction (HFpEF). In a double transgenic rat (dTGR) model of HFpEF, treatment with the sGC stimulator BAY 41-8543 significantly improved survival and reduced cardiac fibrosis, macrophage infiltration, and gap junctions remodeling, highlighting its potential for HFpEF treatment. (Wilck et al., 2018) Taken together, these preclinical data from different models of heart failure etiology suggest that sGC stimulators can be beneficial in the treatment of heart failure in patients. Heart failure (HF) is a serious and terminal stage of various heart diseases with a high incidence rate, and has become the most important CV disease in the 21st century. Studies estimate that the prevalence of heart failure in China will increase by 44% by 2035. (Hao et al., 2019) There is no doubt that HF is a growing global public health and economic burden. Although traditional drugs based on the inhibition of renin-angiotensin-aldosterone and the sympathetic nervous system can delay HF progression and improve clinical symptoms, their morbidity and mortality are increasing globally. Treatment with new drugs is particularly important to further improve the prognosis of patients with HF. So, targeted drugs for the NO/sGC/cGMP signaling pathway may be new selections to the treatment of HF.

sGC stimulators bind to heme-containing sGC and promote the production of cGMP. They can enhance the affinity of sGC even at very low concentrations of NO, thereby affecting normal heart and blood vessel function. (Ignarro et al., 1987; Münzel, 2008) Studies have confirmed that sGC stimulators can treat pulmonary hypertension by lowering pulmonary artery pressure and improving hemodynamics. (Schermuly et al., 2008; Wardle et al., 2016) In addition, some studies have found that sGC stimulators can improve cardiac function and cardiac index (cardiac index, CI). (Mitrovic et al., 2011; Reinke et al., 2015)At present, some preclinical and clinical trials are exploring the effects of new oral sGC stimulators such as riociguat, vericiguat, and praliciguat in the CV field, especially on improving symptoms, reducing mortality and hospitalization rate of HF, and lowering biomarkers of HF in patients with HF. (Follmann et al., 2017; Shea et al., 2020; Udelson et al., 2020) However, due to the heterogeneity of samples, the results are different, and sGC stimulators have different subtypes, it is not clear whether all kinds of drugs can be used to treat HF. Clinical practice still encounters uncertainty of dose selection of sGC stimulators. The existing evidence-based research at home and abroad cannot fully explain the above problems; therefore, clinical individualized treatment remains greatly restricted. With the rapid development of sGC stimulators in CV research, staying abreast of emerging trends and key milestones in the development of relevant knowledge is highly important. Currently, there have been clinical meta-analyses of sGC stimulators for the treatment of HF. For example, Zheng et al. (2018) found that sGC can only improve the quality of life of HF patients, but not reduce mortality; another meta-analysis showed no benefit of vericiguat and riociguat in reducing HF-related hospitalizations or mortality; (Ullah et al., 2020) in addition, a meta-analysis by Moghaddam et al. (2021) showed that sGC stimulators can reduce the rate of HF hospitalization in heart failure with reduced ejection fraction (HFrEF), but not cardiovascular mortality, and there was no significant benefit in HFpEF. In short, although there have been systematic reviews and meta-analyses evaluating the role of sGC agonists in HF, the results are still very different. There are no meta-analyses for HFpEF, and the treatment of HF, especially HFpEF, remains controversial. There have been previous bibliometric analyses of the pharmaceutical area to identify knowledge structures and research trends for a drug in the relevant field, such as the bibliometric analysis of triptolide in treating non–small cell lung cancer, (Yang et al., 2022) and bibliometric study of sodium glucose cotransporter 2 inhibitors in cardiovascular research. (Chen et al., 2020) However, to date, few bibliometric analyses have been performed on the studies of sGC stimulators in the CV field.

Bibliometric analysis has been widely used to organize knowledge structures and explore the development trend of research fields through quantitative analysis of patterns in the scientific literature. This method enables researchers to understand a range of research topics, quickly discover the basic knowledge structure of the research field, explore potential research frontiers and trends, and predict future directions. To the best of our knowledge, no previous bibliometric analysis of sGC stimulators in CV research has been performed. Thus, we aimed to describe the scientific output of CV research on sGC stimulators to determine trends and hotspots to guide researchers’ future work.

## 2 Materials and methods

### 2.1 Data source and search strategies

Data were downloaded from the Science Citation Index-Expanded database of the Web of Science Core Collection (WOSCC) on 3 March 2022. The time span is as follows: the library was built until 31 December 2021. Since the number of articles published in 2022 is not representative of the total number of articles published in 2022 as of the date of retrieval (3 March 2022), so we set a data mining deadline of 31 December 2021. Selection of retrieval methods: subject headings and/or free words were included in the title, keywords, or abstract, and citations were used to search for omissions and fill in the gaps. The search terms used were “Soluble Guanylyl Cyclase*” OR “Cyclase, Soluble Guanylyl*” OR “Guanylyl Cyclase, Soluble*” OR “Cyclase, Soluble Guanylate*” OR “sGC *” OR “Vericiguat*” OR “BAY 1021189*” OR “riociguat*” OR “BAY 63-2521*” OR “BAY-63-2521*” OR “Adempas*” OR “praliciguat*” OR “IW-1973*” AND “heart” OR “*cardi*“. Only original articles and reviews written in English and published from the time of database creation to 2021 were included. Altogether, 1,246 records of data were initially retrieved; Then, applying filters, the literature type is limited to “ARTICLE” or “REVIEW,” and the language of publications is English. We finally obtained 1,212 publications and imported the records into CiteSpace and VOSviewer for analysis.

The inclusion criteria used in this study are as follows: 1) The study is about sGC stimulators in the CV field. 2) The publication period is from 1 January 1992 to 31 December 2021.3) The language is English. 4) The literature type is “ARTICLE” or “REVIEW.”

The exclusion criteria applied in this study are as follows: 1) The types of literature are conference, report, and letters. 2) The literature did not show the research of sGC stimulators in CV field. 3) The language is not English. Since the WOSCC database can only export a maximum of 500 documents at a time, and CiteSpace and VOSviewer software can only recognize data in text format, we initially retrieved 1,226 documents from the WOSCC database and exported them in “.txt” text format three times. Then, rename all downloaded documents to “download_1-500.txt,” “download_501-1000.txt”, “download_1001-1226.txt” respectively. And the reasons why we use the strategies mentioned above are due to the WOS as a famous database that collects authoritative literature all over the world, especially in more medicine and the natural sciences; In additions, the WOSCC is the most frequently used citation database for bibliometric analysis. It can also provide the following information: annual output, authors, journals, institutions, countries/territories, languages, and funds. So, we think that the literature in WOS is representative than PubMed or Scopus. English is a universal language, so we consider that the literature in English is more standard and meaningful than the literature in other languages.

### 2.2 Data collection and analysis

All records retrieved from the WOSCC were downloaded independently by two authors (WYF, AJY) and included the number of annual publication outputs; outputs of countries/regions, institutions, journals, and authors; citation frequency; and the Hirsch index (H-index). The H-index, which indicates that an academic journal or scholar/country/region has published H papers, each of which was cited at least H times, was used to evaluate the scientific impact of an author or country (Hirsch, 2005). Journal Citation Reports (JCR) (Garfield, 1972) 2021 was used to obtain the impact factor (IF) and quartile of a journal category. The document analysis tool of the WOS database was used to calculate the annual volume of articles.

VOSviewer is visualized software based on the Java platform, which is a powerful tool for creating accessible maps using bibliographic data. (van Eck and Waltman, 2010) And VOSviewer1.6.7 was used to create network visualization maps to analyze the collaborative relationships between countries/regions, institutions, and authors of highly co-cited references. In addition, VOSviewer can cluster keywords with high co-occurrence frequency into multiple clusters and color them by time progress simultaneously. Co-occurrence network analysis was used to identify research hotspots and trends. In this study, the “Countries,” “Author” and “Author keywords” were selected as the unit of analysis, the counting method was selected as “Full counting”, the threshold value was selected as “5”; and “Maximum number” was selected as the default setting of “25.” The reason for this setting is that too large number will lead to information noise, while too little number miss the information that needs to be highlighted.

CiteSpace is a visual knowledge mapping bibliometrics tool invented by Chen Meichao of Drexel University based on Java language. (Chen, 2004) And we used CiteSpace 5.8. R3 to perform a co-citation network analysis of journals, references, and their clusters and further construct a timeline view of co-cited reference and keyword clusters, which can identify the rise and period of certain clustering fields. In addition, CiteSpace can capture keywords with strong citations and build visualization maps for all items. Citation bursts are key indicators for identifying emerging trends. Software parameter were set as follows: “Time Slicing” was selected from 1992.01-2021.12 (because the earliest relevant researches were published in 1992). We set the “years per slice” at 1, “Term Source” as “Title,” “Abstract,” “Author Keywords,” “Keywords Plus (ID),” “Term Type” as no selection, and “top N per slice” values as 100; thus, the network map was extracted from the top 100 cited papers in one year per slice. The time slice (years per slice) was selected for 1 year, and the threshold remained in the default state. The network pruning settings were “Pathfinder” and “Pruning sliced networks,” while the others were default settings. The parameter settings were compared repeatedly so that our maps were not too complex and did not miss important nodes. And the analysis results are represented by a visual knowledge graph and corresponding tables. The visualization maps were composed of nodes and connections of different colors. Different colors represent the time range of the input data. The nodes are represented by annual rings, and the rings extending from the inside to the outside represent a gradually increasing time series. The width of the cycle represents the number of citations in the corresponding year. In addition, the size of the nodes represents the total number of citations over the entire period being analyzed. The length and width of the connection represent the intensity of the association and the color of the connection represents the year of the first common citation.

## 3 Results

### 3.1 Publication output and temporal trend

**FIGURE 1:**
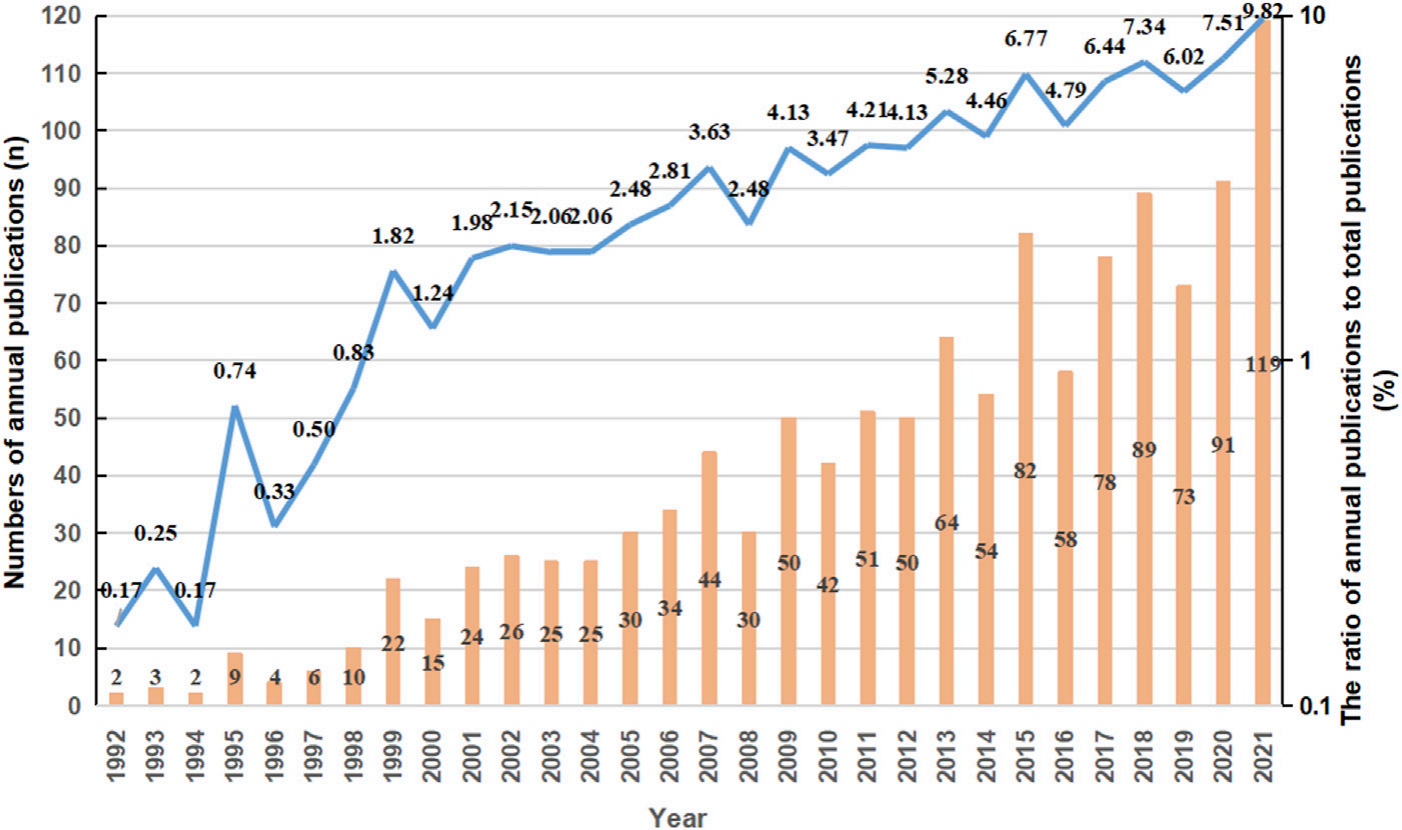
The annual number of publications and trend on sGC stimulators in cardiovascular research between 1992 and 2021.

A total of 1,216 publications met the inclusion criteria. Among them, relevant research first appeared in 1992, and from 1992 to 2021, the output of annual publication has been on the rise, increasing sharply from 2019 to 2021, and the number of publications in 2021 was 119. (Figure 1). It can be seen from Figure 1, the quantities of documents on sGC stimulators in CV field have gone through three stages, namely “Initial”, “Primary Development”” and “Relative Consolidation and Stable development” explained below.

Initial stage (1992-1998): From the first article of sGC stimulators in CV field published in 1992 to the late 1990’s, there were few of related research results in this field, and the maximum annual number of published papers was only ten, which means a complete document system had not yet been formed.

Primary development stage (1999–2009): At this stage, the number of related documents began to rise in volatility, increasing by an average of 2.5 articles per year. It can be considered that the study of sGC stimulators in CV field was initially formed during this period.

Relative Consolidation and Stable development (2010-2021): With the recognition of NO-sGC-cGMP signal pathway in many countries, the research of sGC stimulators in CV field has been paid more and more attention, there had been a relatively stable growth with an average annual growth of 5.5 articles.

This shows that research on sGC stimulators has been a concern for researchers in the CV field. Research enthusiasm has increased in recent years; however, major and difficult problems need to be resolved.

### 3.2 Distribution of country/region and institution

**FIGURE 2:**
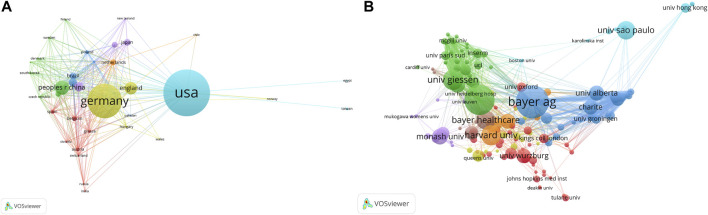
VOSviewer network visualization map of country/regions and institutions involved in sGC situmators in cardiovascular research. **(A)** Collaboration analysis of countries/regions **(B)** Collaboration analysis of institutions.

Countries/regions and research institutions are platforms for researchers to carry out scientific research, providing researchers with financial, equipment, and human support as well as the ownership of scientific research results. By analyzing these results, countries**/**regions and institutions with great influence in this field may be identified. All publications were distributed across 49 countries/regions and 215 institutions. The United States had the highest number of publications in the past 30 years, from 1992 to 2021, with 410 (33.83%) papers, followed by Germany (*n* = 301, 24.83%), the People’s Republic of China (*n* = 128, 10.56%), England (*n* = 81, 6.68%), and Italy (n = 61, 5.03%) (Table 1). Among them, the United States, England and Canada belong to Anglo region, with a total of 535 papers (4.41%); Germany, the Netherlands and Australia are from Germanic Europe region, with a total of 383 papers (3.16%). One of the most co-cited papers by Ghofrani et al. (2013b) was from the Bayer AG Institution in the Germanic Europe region. In additions, China and Japan are from Confucian, with a total of 172 articles (1.42%). Although most of the authors with the most publications come from German institutions, the United States has been the country with the highest number of publications in the past 30 years. This may be related to the earlier initiation of the field in the United States. Moreover, before the emergence of sGC stimulators, Ignarro, a highly co-cited author in the United States, had studied the NO/SGC/cGMP signaling pathway in 1981, and found that NO could relax vascular smooth muscle, and won the Nobel Prize for this. This may have led to a wide range study of sGC stimulators in CV disease in the United States. To investigate international collaborations, we used VOSviewer to construct a network visualization map for publications on CV research, particularly, on sGC stimulators. Since the number of countries satisfied (39 and 35, respectively) at threshold 4 or 6 did not differ much from the number at threshold 5, so we chose the default threshold of 5. At this point, the nodes and links of the cooperative network map was more appropriate, which not only showed most of the major contributing countries, but also the links and cooperation between countries. And Figure 2 shows collaborations among countries/regions publishing more than five papers (35 of 49). Countries/regions with higher co-occurrences were classified using the same color. Country/regions with similar colors, identified as countries/regions with closer cooperative relationships, formed clusters. The widths of the lines represent the magnitude of the collaboration. The United States (410) had the highest total link strength, indicating that it participated in most collaborations with other countries worldwide. The countries/regions that collaborated the most with the United States were Germany, England, China, Italy, and Brazil. The cluster indicated in green was led by the People’s Republic of China in collaboration with the United States, France, the Czech Republic, South Korea, and Denmark.

**TABLE 1:** The top 10 countries/regions in cardiovascular research of sGC stimulators.

Rank	Countries	Affiliated regions	Publications (N)	Percentage (N/1212)	H-index	Total citations	Mean citations
1	United States	Anglo	410	33.83	66	17,314	42.26
2	Germany	Germanic R Europe	301	24.83	67	14,482	46.70
3	Peoples’ Republic of China	Confucian	128	10.56	24	2,744	20.79
4	England	Anglo	81	6.68	36	3,762	32.97
5	Italy	Latin Europe	61	5.03	27	3,721	45.21
6	Brazil	Latin American	55	4.51	21	1,400	21.76
7	Canada	Anglo	44	3.58	26	2,208	30.71
8	Japan	Confucian	44	3.63	18	1,229	20.06
9	Netherlands	Germanic R Europe	43	3.55	20	1,412	27.15
10	Australia	Germanic R Europe	39	3.22	22	1767	35.34

The ten most productive institutions in the relevant research are listed in Table 2. The leading institutions with a focus on sGC stimulation in CV research were Bayer AG (*n* = 67, 5.53%), Hannover Medical School (n = 50,4.13%), University of Giessen (*n* = 44,3.63%), Bayer HealthCare (n = 42,3.47%), and Bayer Pharmaceuticals AG (*n* = 25, 2.06%). To reveal potential collaborations among institutions, we used VOSviewer to conduct a co-authorship analysis in terms of institutions (Figure 2B). Although Duke University in the United States has not reached the top five in the number of articles published, it has close ties with other institutions. Germany’s Bayer AG is the largest contributor, and Bayer and sub-group institutions account for three of the top five institutions (Bayer, 1988). Bayer has 350 branches located in almost all countries and regions worldwide that developed many major drugs, including aspirin, heroin, methadone, ciprofloxacin, and other commonly used and clinically important drugs. Of course, it also includes sGC stimulators and activators. (Evgenov et al., 2006) Comprehensive analysis shows that the top five institutions are all German university institutions and medical research centers. Germany ranks second among high-yielding countries/regions, and works close in cooperation with Duke University, Northwestern University, and Brigham Women’s Hospital. This shows that the United States and Germany are not only countries with the highest output production, but also important countries and institutions that study sGC stimulators in CV research.

**TABLE 2:** The top 10 institutions involved in cardiovascular research of sGC stimulators.

Rank	Institution	Affiliated country	Publications (N)	Percentage (N/1212)	H-index	Total citations	Mean citations
1	Bayer AG	Germany	67	5.53	48	5,972	54.65
2	Hannover Med Sch	Germany	50	4.13	22	1,454	30.64
3	Univ Giessen	Germany	44	3.63	19	1961	52.68
4	Bayer HealthCare	Germany	42	3.47	39	4,334	65.72
5	Bayer Pharma AG	Germany	25	2.06	27	2,422	41.65
6	Univ Sao Paulo	Brazil	23	1.90	15	728	17.75
7	Univ Ghent	Belgium	21	1.73	21	1,286	30.33
8	Harvard Univ	United States	21	1.73	30	3,735	67.33
9	Charite	Germany	20	1.65	18	1,478	41.73
10	Monash Univ	Australia	19	1.57	16	1,223	27.84

### 3.3 Distribution of journal

Bradford’s law is a pattern first described by Samuel C. Bradford in 1934 that estimates the exponentially diminishing returns of extending a search for references in science journals. It is a law that quantitatively describes the concentration-dispersion of professional papers in relevant journals. (Brookes, 1969) After the revision and research of many researchers, it has developed into the famous theory of literature distribution. Each kind of sci-tech periodical belongs to a certain discipline classification, if the sci-tech journal is arranged in decreasing order according to the number of professional papers published in a certain discipline, then the journal can be divided into core area, related area and non-related area. The number of articles in each district is equal, and the number of journals in the core area, related area and non-related area becomes the relationship of 1: n: n^2^.

In our study, 1,212 publications on GC stimulators in CV research were published in 428 academic journals. According to Bradford’s Law, the number of articles in the core area of all literatures were 404, and according to its formula, it can be estimated that there were about 19 or 20 core journals, while the top 10 journals in Table 3 have published 250 articles, accounting for 20.62% of the total, but less than 1/3, indicating that the journals listed in the table were the core journals of sGC stimulators in CV research. The ten most productive and co-cited journals (at least ten citations) are listed in Table 3. *Circulation* (57 publications, 4.70%), which had an impact factor (IF) of 39.918 in 2021, published the most studies in this field, followed by Circulation Research (40, 3.44%), and Proceedings of The National Academy of Sciences of the United States of America (37, 3.05%). The number of articles published in these three journals accounted for 11.07% of all included articles. The journal with the highest IF is New England Journal of Medicine, with an IF of 176.076 in 2021, followed by Nature (69.504) and Circulation (39.918). Among the top 10 journals, Circulation had the highest H-index (27) and total co-citation frequency (2109), but the highest frequently co-cited per article was *Circulation Research* (142.36). According to the 2021 JCR standard, six of the ten most productive journals are in Q1 (the top 25% of the IF distribution), and the rest are in Q2 (between the 50th and 25th percentiles).

**TABLE 3:** Top 10 journals with published papers in cardiovascular research of sGC stimulators.

Rank	Journal	Publications (N)	Percentage (N/1212)	Total citations	Mean citations	H-index	2021-IF	2021JCR best quartile
1	Circulation	57	4.70	2,109	141.27	27	39.918	Q1
2	Circulation Research	40	3.30	1984	142.36	25	23.213	Q1
3	Proceedings of The National Academy of Sciences of the United States of America	37	3.05	813	47.60	25	12.779	Q1
4	Journal of Biological Chemistry	21	1.73	857	61.56	12	5.486	Q2
5	British Journal of Pharmacology	18	1.49	1,420	38.84	21	9.473	Q2
6	American Journal of Physiology-Heart and Circulatory Physiology	17	1.40	1930	34.30	26	5.125	Q2
7	New England Journal of Medicine	16	1.31	742	51.08	13	176.076	Q1
8	Journal of Clinical Investigation	15	1.24	472	19.53	10	19.486	Q1
9	Nature	15	1.24	651	31.38	11	69.504	Q1
10	Journal of the American College of Cardiology	14	1.16	605	40.14	13	27.203	Q1

Note: IF, impact factor; JCR, journal citation report.

### 3.4 Distribution of author

The authors’ scientific research activities in a certain subject area, and the quantity and quality of their publications play an important role in promoting the development of the discipline to some extent. By selecting the authors who have made outstanding contributions in the current research field, and examining their articles, we can judge the current research hotspots in this field. And the Lotka’s law, was developed by American scholar Alfred J. Lotka put forward the empirical law of describing scientific productivity in the 1920s, also known as the “reciprocal square law.” Lotka’s law states that an author who publishes n papers is one n^2^ of an author who publishes 1 paper, and that about 60.79% of authors publish 1 paper in a field. (Schorr, 1975; Coile, 1977) This law reveals for the first time the relationship between the author and the number of articles published, through the statistical discovery of published works, the productive capacity of scientists and technologists, and their contribution to the progress of science and technology and the development of society. In this study, the top 10 authors with the number of publications and citations are listed. According to this quantity, their affiliated institutions can be determined by searching their academic background, and their main literature can be further read, or their colleagues or their students and mentors can be found. More core authors can also be obtained.

A total of 1,294 authors contributed to the cardiovascular field of sGC stimulators in our study. The top ten authors with the highest number of posts and citations are listed in Table 4. Stasch JP published 46 articles, ranking first in the number of publications, followed by Roessig L (20), Sandner P (17), Pueske B, Gruenig E, Brouckaert P, and Hoeper M in fourth place, each publishing 16 articles. In addition, 7 of the 10 authors with the largest number of articles were from Germany, with a total of 159 (13.12%). Two of the 10 authors are currently working with Bayer or its subsidiaries, and the other two worked at the Hannover Medical School in Germany. At the same time, 3 of the 10 highly co-cited authors are from Germany and 3 from the United States. These results demonstrate once again the important contribution of universities or institutions in the United States and Germany to this field.

**TABLE 4:** Top 10 productive authors and co-cited authors in cardiovascular research of sGC stimulators.

Rank	Author	Affiliated institutions/Countries	Publications (N)	Rank	Co-cited author	Affiliated institutions/Countries	Publications (N)
1	Stasch JP	Bayer AG/Germany	46	1	Stasch JP	Bayer AG/Germany	268
2	Roessig L	Bayer Pharma AG/Germany	20	2	Ghofrani HA	Univ Giessen/Germany	222
3	Sandner P	Hannover Med Sch/Germany	17	3	Ignarro LJ	Univ California/United States	167
4	Pieske B	Charite/Germany	16	4	Evgenov OV	Harvard Univ/United States	163
5	Gruenig E	Heidelberg Univ/Germany	16	5	Galie N	Univ Bologna/Italy	156
6	Brouckaert P	Univ Ghent/Belgium	16	6	Friebe A	Univ Wurzburg/Germany	151
7	Hoeper M	Hannover Med Sch/Germany	16	7	Moncada S	Univ Manchester/England	146
8	Rosenkranz S	Univ Cologne/Germany	14	8	Simonneau G	Univ Paris Saclay/France	115
9	Ghofrani H	Univ Giessen/Germany	14	9	Munzel T	Univ Medicine Maine/Germany	114
10	Lam CSP	National Heart Center Singapore	14	10	Gheorghiade M	Northwestern Univ/United States	104

**FIGURE 3:**
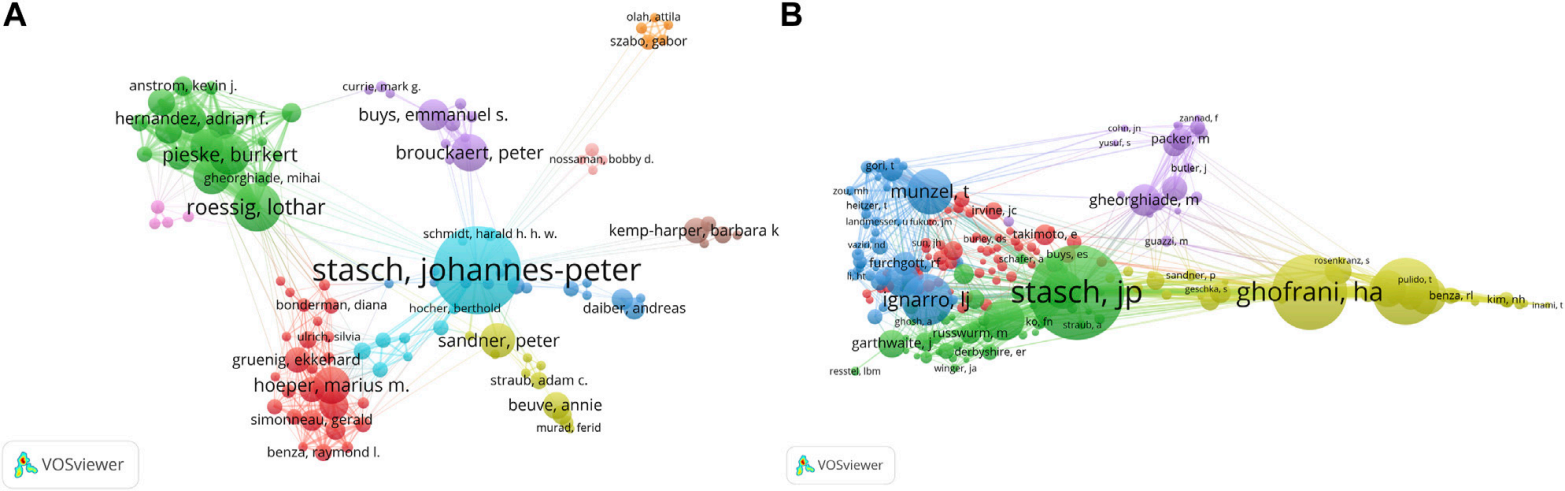
VOSviewer network visualization maps of authors and co-cited authors of the articles related to sGC stimulators in cardiovascular research. **(A)**Map of author Collaboration network analysis **(B)**Map of co-cited authors analysis.

The network visualization maps of the author collaboration network analysis and co-cited author analysis are shown in Figures 3A,B. The sizes of the nodes in the maps are related to the frequency of citations. Eleven authors had frequencies of more than 100. The top five co-cited authors were Stasch JP (268 citations), Ghofrani HA (222 citations), Ignarro LJ (167 citations), Evgenov OV (163 citations), and Galie N (156 citations). Among them, Professor Stasch JP from the Bayer AG institution, a German institution with global influence in the fields of healthcare and agricultural life sciences, is not only the most productive author but also the most frequently co-cited author. As a chemist and pharmacologist, Stasch JP is mainly engaged in cardiovascular and drug research and has previously elucidated the role of the NO/sGC/cGMP cell signaling pathway in the development of cardiovascular diseases. (Stasch et al., 2006) Based on these results, new active ingredients for the treatment of pulmonary hypertension and cardiac dysfunction have been developed, and several highly cited papers have been published in high impact journals, indicating that Professor Stasch JP’s research has a significant influence on sGC stimulators in CV research. Professor Ghofrani HA is the main author evaluating the clinical effects of sGC stimulators in the treatment of CTEPH and PAH. Ignarro LJ was one of the first authors to discover that endothelial cell relaxation factor (EDRF) had the same effect as that of nitric oxide (NO), (Ignarro et al., 1981; Furchgott et al., 1984) and was the winner of the 1998 Nobel Prize in Physiology or Medicine.

### 3.5 Analysis of Co-Cited references

**FIGURE 4:**
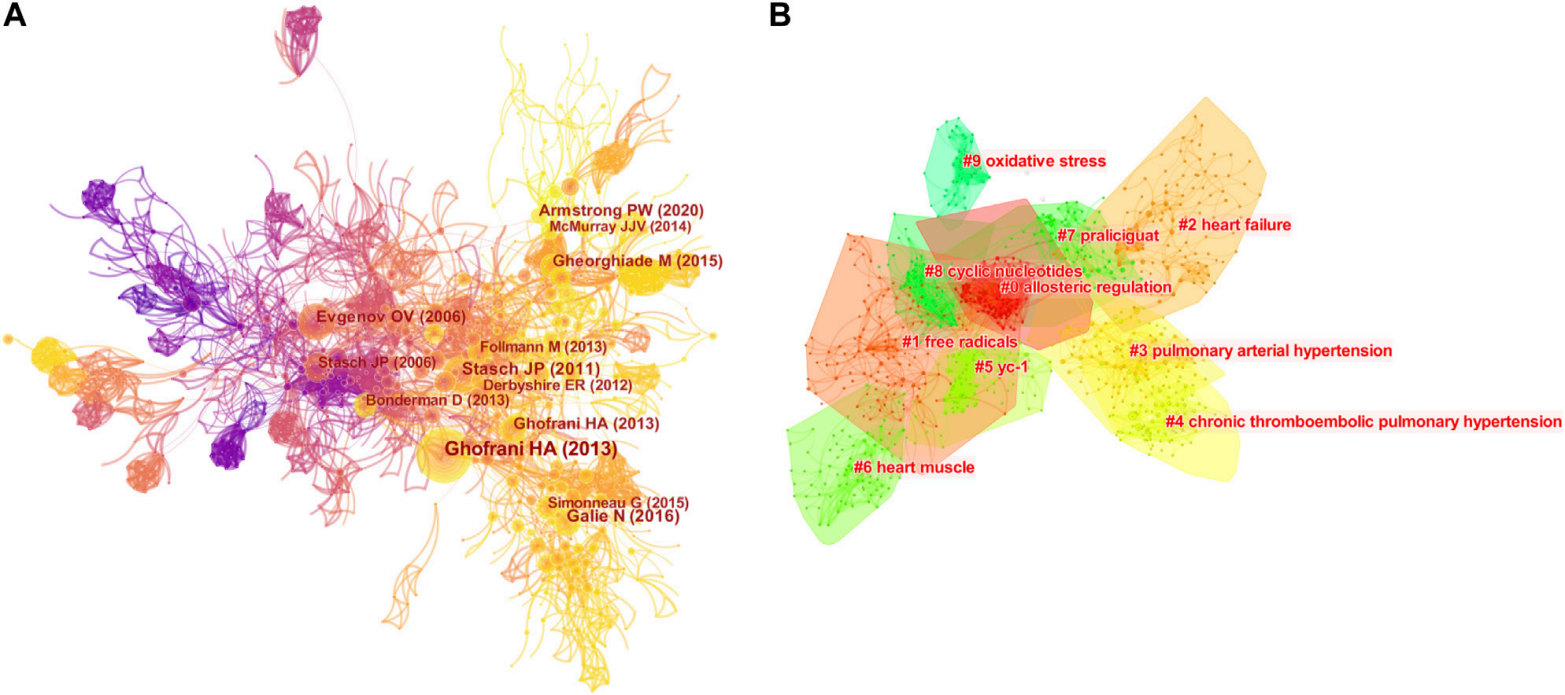
Map analysis of co-cited references to sGC stimulators in cardiovascular research. **(A)** Network map of co-cited references. **(B)** Network map of co-cited reference clusters.

We imported the 1,226 documents into CiteSpace software and obtained a network map of co-cited references with 1709 nodes, 5,953 connections, and a network density of 0.0041 (Figure 4A). The top ten highly cited references are listed in Table 5. Among the highly cited references in the CV research field of sGC stimulators, three papers had a frequency of more than 100 citations. The highest-cited article was published by Ghofrani et al. 2013a on the clinical trial of the sGC stimulator riociguat in the treatment of CTEPH, and the seventh highest-cited reference was also published by the same author, Ghofrani HA, which mainly evaluated the efficacy of riociguat in PAH. (Ghofrani et al., 2013b) These two studies have important implications for the 2013 Food and Drug Administration (FDA) approval of riociguat for CTEPH and PAH. (Stasch and Evgenov, 2013; Dasgupta et al., 2015) The second most highly co-cited reference was the 2015 version of the European Society of Cardiology (ESC) and the European Respiratory Society (ERS) guidelines for the diagnosis and treatment of pulmonary hypertension developed by Galie et al. (2016) and for the first time formally included in the guidelines was the application of riociguat in PAH and CTEPH. The third and eighth highly co-cited articles were all published by Professor Stasch JP, with the highest output production. His articles mainly expounded the mechanism of the NO/sGC/cGMP signal pathway and how the NO receptor can act to dilate diseased blood vessels, (Stasch and Hobbs, 2009) and reviewed soluble guanylate cyclase as a new therapeutic target for cardiopulmonary diseases. (Stasch et al., 2006) Furthermore, the large-scale clinical trial VICTORIA of Armstrong PW is important. (Armstrong et al., 2020a) The trial, conducted in collaboration between the Canadian VIGOUR Center and the Duke Clinical Institution, is a multicenter, parallel, double-blind, randomized placebo-controlled study involving 5,050 patients with HFrEF. The main aim of this study was to assess whether the sGC agonist vericiguat can prolong the time to the first composite endpoint of CV death and hospitalization for patients with HFrEF in the context of standard treatment. The results showed that a median follow-up of 10.8 months reduced the risk of CV death or hospitalization for HF, which was of great significance for FDA approval of vericiguat for symptomatic chronic heart failure (CHF) in 2021, and the first sGC stimulator for treating CHF. Another study by Armstrong et al., the VITALITY-HFpEF trial, also plays an important role in promoting CV research on sGC stimulators. (Armstrong et al., 2020b)

**TABLE 5:** Top 10 co-cited references in cardiovascular research of sGC stimulators from 1992 to 2021.

Rank	References	If	Citations	Year
1	Ghofrani HA, 2013, NEW ENGL J MED, V369, P319, DOI 10.1056/NEJMoa1209657	70.67	169	2013
2	Galie N, 2016, EUR HEART J, V37, P67, DOI 10.1093/eurheartj/ehv317	23.239	166	2016
3	Stasch JP, 2011, CIRCULATION, V123, P2263, DOI 10.1161/CIRCULATIONAHA.110.981,738	23.054	158	2011
4	Evgenov OV, 2006, NAT REV DRUG DISCOV, V5, P755, DOI 10.1038/nrd2038	57.618	98	2006
5	Armstrong PW, 2020, NEW ENGL J MED, V382, P1883, DOI 10.1056/NEJMoa1915928	70.67	86	2020
6	Gheorghiade M, 2015, JAMA-J AM MED ASSOC, V314, P2251, DOI 10.1001/jama.2015.15734	51.234	79	2015
7	Ghofrani HA, 2013, NEW ENGL J MED, V369, P330, DOI 10.1056/NEJMoa1209655	70.67	75	2013
8	Stasch JP, 2006, J CLIN INVEST, V116, P2552, DOI 10.1172/JCI28371	12.282	72	2006
9	Bonderman D, 2013, CIRCULATION, V128, P502, DOI 10.1161/CIRCULATIONAHA.113.001458	23.054	69	2013
10	Follmann M, 2013, ANGEW CHEM INT EDIT, V52, P9442, DOI 10.1002/anie.201302588	12.257	68	2013

We also constructed a network map to visualize the key clusters of co-cited references (Figure 4B). The modularity Q score of the clustering map was 0.9243 and the mean silhouette value was 0.8261, indicating a stable network structure. There were ten major clusters, among which cluster #0, labelled “allosteric regulation” was the largest cluster, consisting of 45 references, followed by “free radicals” (cluster #1), “heart failure” (cluster #2), “pulmonary arterial hypertension” (cluster #3), and chronic thromboembolic pulmonary hypertension” (cluster #4).

**FIGURE 5:**
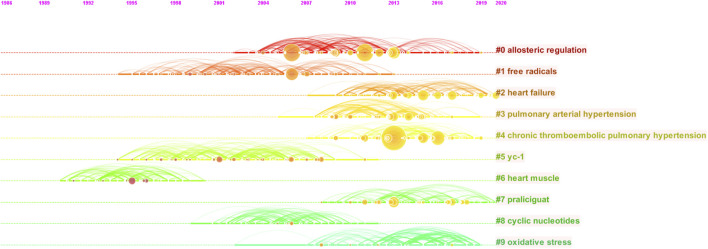
Timeline view of co-cited references related to sGC stimulators in cardiovascular research.

Furthermore, we performed a timeline analysis of the co-cited references (Figure 5). The map shows the relationship and time span between the clusters and references in each cluster. The more documents in the cluster, the more important it is to represent the clustering field. Most articles were published after 2006, consistent with the results shown in Figure 1. “Heart muscle” (cluster #6) and “free radicals” (cluster #1) were relatively early research hotspots. Cluster #4 (“chronic thromboembolic pulmonary hypertension”), with the largest nodes and the warmest color, contained the most publications, indicating that this cluster was the main direction of cardiovascular research on sGC stimulators around 2013. HF (cluster #2) was the main cluster from 2016 to the present; the color continued to be warm, and the node size did not change much, indicating that HF is the primary research hotspot of sGC stimulators in CV research.

### 3.6 Analysis of keyword

Keywords in the literature condense research content. Similarly, keywords in a field also reflect the research direction of the field to a large extent. Some people believe that an increase in the number of keyword citations or an increase in the frequency of keyword occurrence within a certain period is an indicator for evaluating the most cutting-edge topics or emerging trends. Therefore, keyword analysis of references in a certain field can determine current research hotspots and predict the development trend of future research hotspots through the frequency of keyword occurrences.

#### 3.6.1 Analysis of keyword Co-Occurrence and clusters

**FIGURE 6:**
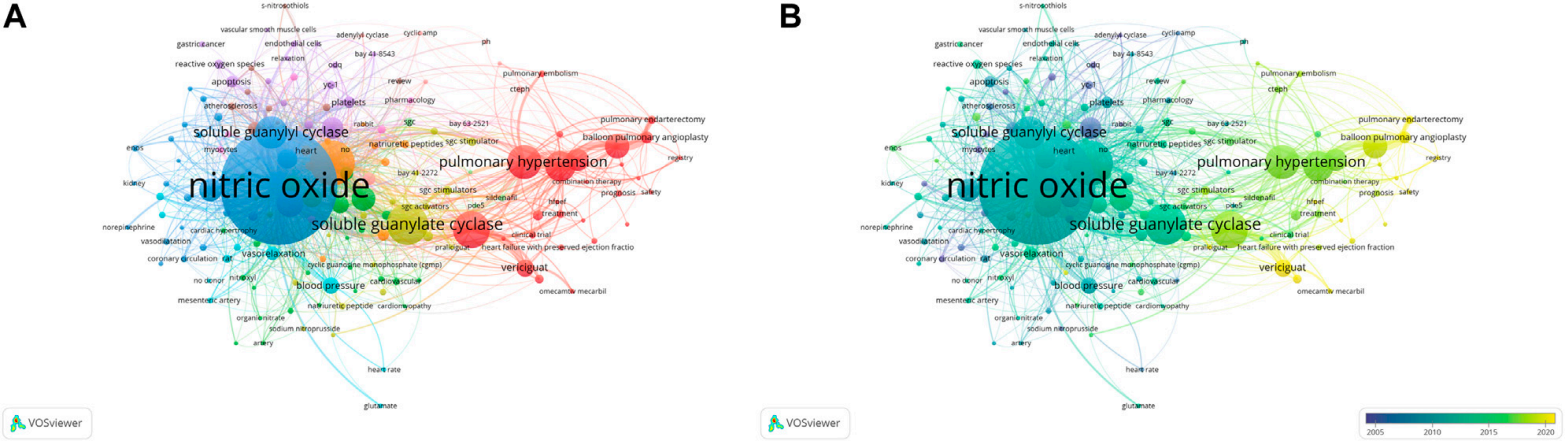
Analysis of keywords in publications related to sGC stimulators in cardiovascular research from 1992 to 2021 **(A)** VOSviewer network visualization map of co-occurring keywords. **(B)** VOSviewer overlay visualization of co-occurring keywords by time.

We used VOSviewer to create a knowledge map of keyword co-occurrence with 159 terms (defined as terms that occurred more than five times) after combining the synonyms into two clusters (Figure 6A). Because when the threshold was selected “4,” 215 keywords met the conditions, and the visualization map was somewhat mixed; while when the threshold was set to “6,” 122 keywords meet the value, and the map was slightly simple; So we use threshold 5. At this time, there were 159 keywords to meet, the result was clear, and different branches of the field can be seen. The size of the circle represents the occurrence frequency of the keyword; the larger the circle, the more frequently it appears. Figure 6B shows an overlay visualization of the keywords over time. The purple, blue, green, and yellow colors on the time course correspond to the appearance of keywords over the average time, from early years to recent years. Among them, the keyword “nitric oxide” appeared the most frequently 689) and appeared earlier. Other high-frequency keywords were “soluble guanylate cyclase” (320), “pulmonary hypertension” (266), “cGMP” (204) and “heart failure” (168). Moreover, the appearance time of keywords such as HF, riociguat, CTPH, and PAH is relatively recent, indicating that the content of these keywords has become a research hotspot in recent years.

#### 3.6.2 Analysis of keywords clustering timeline view

**FIGURE 7:**
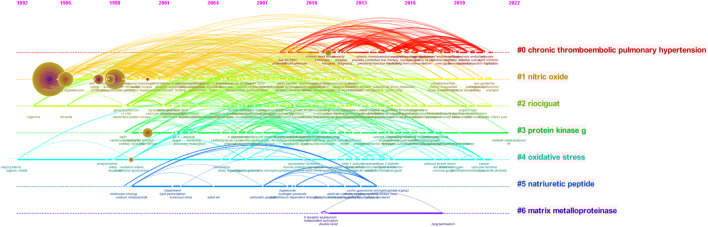
Timeline view of keyword clustering related to sGC stimulators in cardiovascular research.

CiteSpace software was used to merge the same keywords to obtain a timeline view of keywords (Figure 7). Cluster #1 (nitric oxide) has the most references and the largest time span, and related articles were published from 1992 to 2021, indicating that the NO-related mechanism is of great significance to the emergence and development of this field. Cluster #0 (CTEPH) has the warmest color, with many research results. Attention has been high from around 2007 to 2021, and it is expected that related research will continue in the future. Generally, the specific fields represented by clusters #0–3 have a larger time span and more research results.

#### 3.6.3 Analysis of burst keywords

**FIGURE 8:**
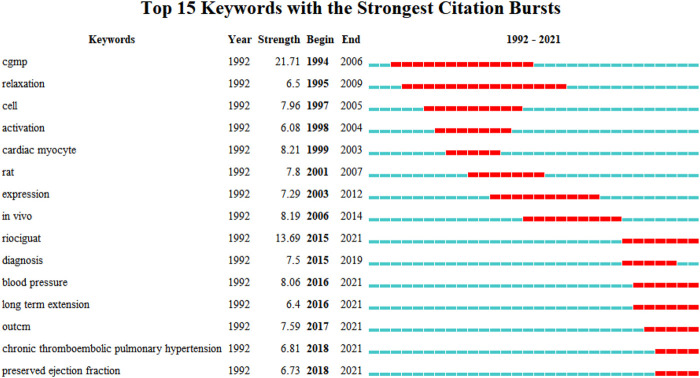
Top 15 keywords with the strongest citation bursts related to sGC stimulators in cardiovascular research.

Keywords with high prominence rates represent a new discovery or turning point in the development of the discipline, have been widely studied in a short time, represent the frontiers in a field, and can identify the hotspots of future research and guide the development of the discipline. We used CiteSpace to detect burst keywords to determine hotspots and research frontiers over time. The results showed that the keyword burst had a year correlation (Figure 8); that is, different sGC stimulators had different periods of research hotspots in the cardiovascular field. The map shows the top 15 keywords with the strongest citation bursts from 1992 to 2021. The red bar indicates that the keyword was frequently cited and the green bar indicates that the keyword was rarely cited. In the early days (1994-1999), “cGMP,” “relaxation,” “cell” “activation,” and “cardiac myocyte” appeared, in the mid-term (2001–2006), “rat,” “expression” and “in vivo” appeared, and in the late stage (from 2015 to the present), “riociguat,” “diagnosis,” “blood pressure,” “long term extension,” “outcome,” “chronic thromboembolic pulmonary hypertension” and “preserved ejection fraction” appeared. Among them, the keyword cGMP, which appeared in 1994, had a burst strength of 21.71, indicating that “cGMP” received the most attention and research from 1994 to 2006. Secondly, the burst strength of the key words "riociguat" highlighted since 2015 was 13.69 and lasting for about five years along with “blood pressure,” “outcome,” “chronic thromboembolic pulmonary hypertension,” “preserved ejection fraction” and “heart failure,” were still obvious in 2021. It was suggested that “blood pressure,” “outcome,” “chronic thromboembolic pulmonary hypertension” and “ejection fraction retention” may represent important content and frontiers in the cardiovascular research field of sGC stimulators and may still be a hot topic for research now and in the coming years.

## 4 Discussion

The NO/sGC/cGMP signaling pathway was a milestone in the elucidation of the intracellular signal transfer pathways regulated by cyclic nucleotides. (Sandner et al., 2021) After discovering that EDRF is the same as NO and that NO can bind to and stimulate sGC, subsequent studies have also provided reliable evidence for the key role of NO/sGC/cGMP signaling. (Ignarro et al., 1987; Murad, 1998) In mammals, there are two key types of guanylate cyclases (GCs), classified according to localization of enzymes in the cell. The first is the guanylate cyclase-coupled receptor or membrane-bound guanylate cyclase (mGC), which functions as a receptor for natriuretic peptides. The second pathway is completely intracellular and serves as a receptor for nitric oxide (NO). (Boerrigter et al., 2009) GC is the intracellular receptor of NO and represents a widely distributed family of enzymes that convert guanosine triphosphate (GTP) to the second-messenger molecule cGMP. (Evgenov et al., 2006) Under physiological conditions, NO-induced sGC stimulation leads to the production of large amounts of cGMP, which regulates the activity of different downstream targets, including cGMP-regulated protein kinase (PKG) and cGMP-regulated ion channels (CNGCs) such as potassium channels. These cGMP downstream targets, especially PKG1 and PKG2, can phosphorylate a wider range of downstream molecules such as regulatory myosin phosphatase (MYPT1) and vasodilator-specific protein (VASP). (Hofmann and Wegener, 2013) The degradation of cGMP is mainly carried out by members of the phosphodiesterase (PDE) superfamily. (Hofmann et al., 2006) The PDEs expressed in cardiomyocytes are PDE1, PDE2, PDE3, PDE5, and PDE9. (Bender and Beavo, 2006) PDE2 and PDE3 are highly expressed in cardiomyocytes and both are present in vascular smooth muscle cells. The role of PDE2 in regulating cardiac function has been well-documented in previous reports. (Fischmeister et al., 2005) In human cardiac myocytes, PDE2 has been shown to regulate cardiac L-type calcium channels, whereas PDE3 is involved in the regulation of cardiac contractility. HF is associated with decreased levels of PDE3, and its low expression leads to cardiomyocyte apoptosis and arrhythmia. (Ding et al., 2005) PDE1 and PDE5 are expressed at lower levels in the heart and mainly in the cytosol. PDE5, a known regulator of vascular smooth muscle contraction, is characterized by relative specificity for cGMP hydrolysis at low substrate levels and exists at the high-affinity binding site of cGMP. (Baillie et al., 2019) Although expression is low in the myocardium, it is worth noting that in the PDE family, PDE9 may have the highest affinity for cGMP, especially in cardiovascular disease, where PDE9A expression is upregulated by cardiac hypertrophy and HF. (Lee et al., 2015) To date, there have been many reports on PDEs and their target drugs.

Moreover, the NO/sGC/cGMP pathway plays an important role in various physiological processes such as platelet aggregation, smooth muscle relaxation, and neurotransmitter transmission. Among them, NO nitrogen-driven cGMP regulates various cellular and tissue functions and plays a key role in maintaining homeostasis of the internal environment, especially in the cardiovascular system. In addition, sGC stimulators have a dual mode of action, which can directly stimulate the native form of the enzyme independent of NO, and they are also able to sensitize sGC to low levels of NO by stabilizing NO–sGC binding. Although previous studies have reported the research status and progress of sGC stimulators in the CV field, most are textual descriptions, and there has been no bibliometric analysis.

In this study, CiteSpace and VOSviewer software were used to analyze a large amount of high-quality data on CV research of sGC stimulators in WOSCC. Through a series of visual maps, we analyzed the potential dynamic mechanism of the discipline and explored the frontiers of discipline development, which showed the main research institutions and leaders in this field and demonstrated the hotspots and shortcomings of sGC stimulators in cardiovascular research, so as to provide a reference for further research.

Through the analysis of the cooperation map of countries/regions and institutions, it was concluded that the high-yielding countries/regions or institutions are mainly institutions, universities, or medical and pharmaceutical research institutes in Germany and the United States. Among them, the Bayer institution, which focused on related research earlier than researchers from other regions of the world, made a significant leading contribution to the earliest discovery of NO independent sGC stimulators. In 1994, researchers at Bayer conducted a screening trial to identify substances that can induce an increase in NO synthesis and stimulate sGC in pig endothelial cells. These studies involved measuring cGMP levels using radioimmunoassay and accidentally found sGC stimulants that did not depend on NO. (Schmidt and Walter, 1994) In addition, Bayer has made great contributions to the development of sGC-stimulating drugs and cardiovascular indications, and the Heart Center of the University of Hannover, Germany, mainly studies the relevant mechanisms of the NO/sGC/cGMP signaling pathway in CV disease.

Journal analysis found that the journal with the most published articles on CV studies of sGC stimulators is Circulation, which belongs to JCR Peripheral Vascular Disease Zone 1, Heart and Cardiovascular system Zone 1, with an IF of 26.69 in 2021. Moreover, two of the top ten most frequently cited documents were published in *Circulation*. Overall, the journal has a higher average IF, H-index, and international influence of related references. New England Journal of Medicine (NEJM) has the highest IF (91.243 in 2021), and three of the top ten highest cited papers have been published in the journal.

In addition, Circulation Research, Journal of the American College of Cardiology (JACC), The Journal of the American Medical Association (JAMA), and European Heart Journal (ESC) are not only the main journals collecting cardiovascular-related research on sGC stimulants but also important journals in the field of CV medicine. Among them, the ESC also publishes guidelines for the diagnosis and treatment of hypertension, pulmonary hypertension, and HF. (Galiè et al., 2016)

The IF and H- indexes of these journals are above ten, and the JCR zone is essentially the Q1 zone. The main authors, institutions, and highly co-cited papers on sGC stimulators in cardiovascular research are also mainly published in the above-mentioned journals, with high academic influence. These journals were thus recognized as basic research resources and play an important role in this research field.

Through analysis of authors, reference cooperation maps, and co-citations, it was found that the authors with the most publications and citations were Professor JP Stasch, and he had a close cooperative relationship with Professor P Sandner of the University of Hanover in Germany and Professor HA Ghofrani of the University of Giessen in Germany. JS Shah has played a leading role in the discovery and development of sGC stimulators and activators, and has developed a variety of compounds, including riociguat (Mittendorf et al., 2009; Stasch et al., 2011), two of which occupy the third and eighth place in the highly co-cited references of sCG stimulators in cardiovascular research. As a pharmacologist and physiologist, P Sandner is known as the chief scientist in sGC pharmacology and cardiovascular research. Currently, he is mainly devoted to the application of new sGC stimulators and activators in cardiovascular and other diseases, such as arteriosclerosis, benign prostatic hyperplasia, kidney disease, and systemic sclerosis (SSc), a rare autoimmune disease. (Petzuch et al., 2022; Zabbarova et al., 2022) HA Ghofrani mainly studied the clinical application of sGC stimulators in CTEPH and PAH (Ghofrani et al., 2013a; Ghofrani et al., 2013b). In patients with PAH, riociguat significantly improved exercise capacity and secondary efficacy endpoints, including improvement in 6-min walk distance, pulmonary vascular resistance, NT-proBNP levels, WHO functional class, time to clinical deterioration, and Borg dyspnea score. Another highly cited study found similar results in patients with CTEPH, finding that riociguat improved the 6-min walk test, decreased pulmonary vascular resistance and NT-proBNP levels, and improved life quality in patients with CTEPH. Corresponding to the co-citation analysis of the references, two of the top ten highly cited papers are HA Ghofrani’s research on CTEPH and PAH. The study and its clinical trial results were of great significance for the official approval of riociguat for the treatment of CTEPH and PAH by the FDA in 2013 and the ESC/ERC in 2015. In addition, R Furchgott found that EDRF was identical to NO, and found that NO can bind and stimulate sGC. Ignarro LJ, one of the highly co-cited authors, derived a similar conclusion in 1981. Both researchers presented evidence at conferences during 1986, demonstrating that EDRF is in fact NO. In 1998, the Nobel Prize in Physiology or Medicine was jointly awarded to Ignarro and Furchgott for their discovery of the mechanism of action of NO in the body. This is of great significance to the mechanism of the NO/sGC/cGMP signaling pathway and subsequent related research. As shown in the timeline view of the co-cited references, cluster #4 (“chronic thromboembolic pulmonary hypertension”), with the warmest color and largest nodes scattered on the timeline, indicated that it was the focus of the research process. Cluster #1 (“heart failure”) contained numerous hotspot nodes with red rings, indicating that it was the most recently formed cluster and the most popular research hotspot and direction. The fifth most highly cited literature, the VICTORIA trial, (Armstrong et al., 2020a) is a large randomized controlled study on HF. The VICTORIA study was a multi-center, parallel, double-blind, randomized, placebo-controlled, large-scale study. The trial included 5,050 patients with HFrEF, and was designed to assess whether vericiguat reduces the incidence of heart failure hospitalization or CV death in patients with HFrEF in the context of standard care. Its results showed that vericiguat significantly reduced the risk of the composite endpoint of CV death and hospitalization for heart failure, as well as HF hospitalization risk compared with placebo. However, CV mortality was almost the same in the two groups, indicating that the primary outcome was driven by a lower rate of HF hospitalizations in the vericiguat group. Based on the results of the VICTORIA Phase III clinical trial, in 2021 the FDA approved the vericiguat for symptomatic high-risk patients with chronic HFrEF to reduce their risk of CV events. Moreover, another study on HFpEF by the same author have completed, VITALITY-HFpEF, (Armstrong et al., 2020b) which was a phase IIb, randomized, double-blind, placebo-controlled multicenter trial, designed to assess the effect of vericiguat on the Kansas City Cardiomyopathy Questionnaire (KCCQ) score in patients with HFpEF. The results showed that compared with placebo, vericiguat 15  mg d^−1^ or 10 mg d^−1^ treatment for 24 weeks did not improve the KCCQ score. Although the results did not show a clear benefit of sGC stimulators on HFpEF, it also indicated that related research is a research hotspot of sGC stimulators in the field of CV. Therefore, future studies should focus on the potential therapeutic efficacies of sGC stimulators and their application in HF, as well as the CV effects of other new sGC stimulators, with more high-quality basic research conducted to further clarify the potential related mechanisms.

## 4 Limitations

To the best of our knowledge, this is the first bibliometric study to focus on the development and trends of CV research on sGC stimulators using a scientometric method. However, our study had several limitations. First, bibliometric information related to sGC stimulators in CV research was extracted from the Web of Science Core Collection database, and it is possible that some articles worthwhile not included in this database were excluded from our study. However, we consider the WoSCC database a reliable service for publications and citations, although it may include fewer documents and journals than that of other databases such as Google Scholar or Scopus. Second, the search criteria were limited to articles written in English. This criterion may have led to the exclusion of high-impact articles written in other languages. Third, limited terminology was used in the publication search strategy, which might have led to data bias because the search strategy could not identify all relevant studies in this field.

## 5 Conclusions

In conclusion, this study explored the research status, hotspots, and development trends in the cardiovascular field of sGC stimulators over the past 30 years using bibliometric methods and CiteSpace and VOSviewer visual analysis software. Since 2013, a growing body of research has been conducted to confirm the medical value of sGC stimulators in CV disease. The United States contributes the most to the field, but Germany’s Bayer is the most productive institution and plays an important role in the network of cooperation. The most productive and co-cited journal was *Circulation*, and the most productive author was Stasch JP. “Preserved ejection fraction” may be the most recent research Frontier. Most studies have focused on clinical trial outcomes such as CV death, HF hospitalization, and other cardiovascular outcome events. The therapeutic effect of sGC stimulators in HF, especially HFpEF, may soon become a research hotspot and should be closely monitored.

## Data Availability

The original contributions presented in the study are included in the article/Supplementary Material, further inquiries can be directed to the corresponding author.

## References

[B1] Armstrong P. W. Pieske B. Anstrom K. J. Ezekowitz J. Hernandez A. F. Butler J. (2020a). Vericiguat in patients with heart failure and reduced ejection fraction. N. Engl. J. Med. 382 (20), 1883–1893. 10.1056/NEJMoa1915928 32222134

[B2] Armstrong P. W. Lam C. S. P. Anstrom K. J. Ezekowitz J. Hernandez A. F. O'Connor C. M. (2020b). Effect of vericiguat vs placebo on quality of life in patients with heart failure and preserved ejection fraction: The VITALITY-HFpEF randomized clinical trial. JAMA 324 (15), 1512–1521. 10.1001/jama.2020.15922 33079152 PMC7576403

[B3] Baillie G. S. Tejeda G. S. Kelly M. P. (2019). Therapeutic targeting of 3', 5'-cyclic nucleotide phosphodiesterases: Inhibition and beyond. Nat. Rev. Drug Discov. 18 (10), 770–796. 10.1038/s41573-019-0033-4 31388135 PMC6773486

[B4] Bayer A. G. (1988). Bayer [M]. Bayer AG.

[B5] Bender A. T. Beavo J. A. (2006). Cyclic nucleotide phosphodiesterases: Molecular regulation to clinical use. Pharmacol. Rev. 58 (3), 488–520. 10.1124/pr.58.3.5 16968949

[B6] Bice J. S. Keim Y. Stasch J. P. Baxter G. F. (2014). NO-independent stimulation or activation of soluble guanylyl cyclase during early reperfusion limits infarct size Cardiovasc. Res. 101 (2), 220–228. 10.1093/cvr/cvt257 24259501 PMC3896250

[B7] Boerrigter G. Lapp H. Burnett J. C. (2009). Modulation of cGMP in heart failure: A new therapeutic paradigm. Handb. Exp. Pharmacol. 191, 485–506. 10.1007/978-3-540-68964-5_21 PMC3835600 19089342

[B8] Brookes B. C. (1969). Bradford's law and the bibliography of science. Nature 224 (5223), 953–956. 10.1038/224953a0 4902657

[B9] Chen C. (2004). Searching for intellectual turning points: Progressive knowledge domain visualization. Proc. Natl. Acad. Sci. U. S. A. 101, 5303–5310. 10.1073/pnas.0307513100 14724295 PMC387312

[B10] Chen L. Ma S. Hu D. Lin H. Zhu Y. Chen K. (2020). Bibliometric study of sodium glucose cotransporter 2 inhibitors in cardiovascular research. Front. Pharmacol. 11, 561494. 10.3389/fphar.2020.561494 33041801 PMC7522576

[B11] Coile R. C. (1977). Lotka's frequency distribution of scientific productivity. J. Am. Soc. Inf. Sci. 28 (6), 366–370. 10.1002/asi.4630280610

[B12] Dasgupta A. Bowman L. D'Arsigny C. L. Archer S. L. (2015). Soluble guanylate cyclase: A new therapeutic target for pulmonary arterial hypertension and chronic thromboembolic pulmonary hypertension. Clin. Pharmacol. Ther. 97 (1), 88–102. 10.1002/cpt.10 25670386 PMC4325399

[B13] Ding B. Abe J. I. Wei H. Huang Q. Walsh R. A. Molina C. A. (2005). Functional role of phosphodiesterase 3 in cardiomyocyte apoptosis: Implication in heart failure. Circulation 111 (19), 2469–2476. 10.1161/01.CIR.0000165128.39715.87 15867171 PMC4108189

[B14] Dumitrascu R. Weissmann N. Ghofrani H. A. Dony E. Beuerlein K. Schmidt H. (2006). Activation of soluble guanylate cyclase reverses experimental pulmonary hypertension and vascular remodeling. Circulation 113 (2), 286–295. 10.1161/CIRCULATIONAHA.105.581405 16391154

[B15] Evgenov O. V. Pacher P. Schmidt P. M. Hasko G. Schmidt H. H. H. W. Stasch J. P. (2006). NO-Independent stimulators and activators of soluble guanylate cyclase: Discovery and therapeutic potential. Nat. Rev. Drug Discov. 5 (9), 755–768. 10.1038/nrd2038 16955067 PMC2225477

[B16] Fischmeister R. Castro L. Abi-Gerges A. Rochais F. Vandecasteele G. (2005). Species- and tissue-dependent effects of NO and cyclic GMP on cardiac ion channels. Comp. Biochem. Physiol. A Mol. Integr. Physiol. 142 (2), 136–143. 10.1016/j.cbpb.2005.04.012 15927494

[B17] Follmann M. Ackerstaff J. Redlich G. Wunder F. Lang D. Kern A. (2017). Discovery of the soluble guanylate cyclase stimulator vericiguat (BAY 1021189) for the treatment of chronic heart failure. J. Med. Chem. 60 (12), 5146–5161. 10.1021/acs.jmedchem.7b00449 28557445

[B18] Furchgott R. F. Cherry P. D. Zawadzki J. V. JothiananDan D. (1984). Endothelial cells as mediators of vasodilation of arteries. J. Cardiovasc. Pharmacol. 6, S336–S343. 10.1097/00005344-198406002-00008 6206342

[B19] Galiè N. Humbert M. Vachiery J. L. Gibbs S. Lang I. Torbicki A. (2016). 2015 ESC/ERS guidelines for the diagnosis and treatment of pulmonary hypertension: The joint task force for the diagnosis and treatment of pulmonary hypertension of the European society of Cardiology (ESC) and the European respiratory society (ERS): Endorsed by: Association for European paediatric and congenital Cardiology (AEPC), international society for heart and lung transplantation (ISHLT). Eur. Heart J. 37 (1), 67–119. 10.1093/eurheartj/ehv317 26320113

[B20] Garfield E. (1972). Citation analysis as a tool in journal evaluation. Sci. Am. Assoc. Adv. Sci. 178 (4060), 471–479. 10.1126/science.178.4060.471 5079701

[B21] Ghofrani H. A. D'Armini A. M. Grimminger F. Hoeper M. M. Jansa P. Kim N. H. (2013a). Riociguat for the treatment of chronic thromboembolic pulmonary hypertension. N. Engl. J. Med. 369 (4), 319–329. 10.1056/NEJMoa1209657 23883377

[B22] Ghofrani H. A. Galiè N. Grimminger F. Grunig E. Humbert M. Jing Z. C. (2013b). Riociguat for the treatment of pulmonary arterial hypertension. N. Engl. J. Med. 369 (4), 330–340. 10.1056/NEJMoa1209655 23883378

[B23] Hao G. Wang X. Chen Z. Zhang L. Zhang Y. Wei B. (2019). Prevalence of heart failure and left ventricular dysfunction in China: The China hypertension survey, 2012-2015. Eur. J. Heart Fail. 21 (11), 1329–1337. 10.1002/ejhf.1629 31746111

[B24] Hirsch J. E. (2005). An index to quantify an individual's scientific research output. Proc. Natl. Acad. Sci. U. S. A. 102 (46), 16569–16572. 10.1073/pnas.0507655102 16275915 PMC1283832

[B25] Hofmann F. Feil R. Kleppisch T. Schlossmann J. (2006). Function of cGMP-dependent protein kinases as revealed by gene deletion. Physiol. Rev. 86 (1), 1–23. 10.1152/physrev.00015.2005 16371594

[B26] Hofmann F. Wegener J. W. (2013). cGMP-dependent protein kinases (cGK). Methods Mol. Biol. 1020, 17–50. 10.1007/978-1-62703-459-3_2 23709024

[B27] Ignarro L. J. Byrns R. E. Wood K. S. (1987). Endothelium-dependent modulation of cGMP levels and intrinsic smooth muscle tone in isolated bovine intrapulmonary artery and vein. Circ. Res. 60 (1), 82–92. 10.1161/01.res.60.1.82 3032474

[B28] Ignarro L. J. Lippton H. Edwards J. C. Baricos W. H. Hyman A. L. Kadowitz P. J. (1981). Mechanism of vascular smooth muscle relaxation by organic nitrates, nitrites, nitroprusside and nitric oxide: Evidence for the involvement of S-nitrosothiols as active intermediates. J. Pharmacol. Exp. Ther. 218 (3), 739–749. 6115052

[B29] Irvine J. C. Ganthavee V. Love J. E. Alexander A. E. Horowitz J. D. Stasch J. P. (2012). The soluble guanylyl cyclase activator bay 58-2667 selectively limits cardiomyocyte hypertrophy. PLoS One 7 (11), e44481. 10.1371/journal.pone.0044481 23144773 PMC3492396

[B30] Lang M. Kojonazarov B. Tian X. Kalymbetov A. Weissmann N. Grimminger F. (2012). The soluble guanylate cyclase stimulator riociguat ameliorates pulmonary hypertension induced by hypoxia and SU5416 in rats. PLoS One 7 (8), e43433. 10.1371/journal.pone.0043433 22912874 PMC3422306

[B31] Lee D. I. Zhu G. Sasaki T. Cho G. S. Hamdani N. Holewinski R. (2015). Phosphodiesterase 9A controls nitric-oxide-independent cGMP and hypertrophic heart disease. Nature 519 (7544), 472–476. 10.1038/nature14332 25799991 PMC4376609

[B32] Mátyás C. Németh B. T. Oláh A. Hidi L. Birtalan E. Kellermayer D. (2015). The soluble guanylate cyclase activator cinaciguat prevents cardiac dysfunction in a rat model of type-1 diabetes mellitus. Cardiovasc. Diabetol. 14, 145. 10.1186/s12933-015-0309-x 26520063 PMC4628236

[B33] Methner C. Buonincontri G. Hu C. H. Vujic A. Kretschmer A. Sawiak S. (2013). Riociguat reduces infarct size and post-infarct heart failure in mouse hearts: Insights from MRI/PET imaging. PLoS One 8 (12), e83910. 10.1371/journal.pone.0083910 24391843 PMC3877128

[B34] Mitrovic V. Jovanovic A. Lehinant S. (2011). Soluble guanylate cyclase modulators in heart failure. Curr. Heart Fail. Rep. 8 (1), 38–44. 10.1007/s11897-010-0045-1 21207207

[B35] Mittendorf J. Weigand S. Alonso-Alija C. Bischoff E. Feurer A. Gerisch M. (2009). Discovery of riociguat (BAY 63-2521): A potent, oral stimulator of soluble guanylate cyclase for the treatment of pulmonary hypertension. ChemMedChem 4 (5), 853–865. 10.1002/cmdc.200900014 19263460

[B36] Moghaddam N. Malhi N. Toma M. (2021). Impact of oral soluble guanylate cyclase stimulators in heart failure: A systematic review and meta-analysis of randomized controlled trials. Am. Heart J. 241, 74–82. 10.1016/j.ahj.2021.07.003 34283990

[B37] Münzel T. (2008). [Recent findings on nitrates: Their action, bioactivation and development of tolerance]. Dtsch. Med. Wochenschr. 133 (44), 2277–2282. 10.1055/s-0028-1091272 18946854

[B38] Murad F. (1998). Nitric oxide signaling: Would you believe that a simple free radical could be a second messenger, autacoid, paracrine substance, neurotransmitter, and hormone? [J]. Recent Prog. Horm. Res. 53, 43–59. discussion 59-60. 9769702

[B39] Petzuch B. Benardeau A. Hofmeister L. Meyer J. Hartmann E. Pavkovic M. (2022). Urinary miRNA profiles in chronic kidney injury - benefits of extracellular vesicle enrichment and miRNAs as potential biomarkers for renal fibrosis, glomerular injury and endothelial dysfunction [J]. Toxicol. Sci. 187 (1), 35–50. 10.1093/toxsci/kfac028 35244176

[B40] Reinke Y. Gross S. Eckerle L. G. Hertrich I. Busch M. Busch R. (2015). The soluble guanylate cyclase stimulator riociguat and the soluble guanylate cyclase activator cinaciguat exert no direct effects on contractility and relaxation of cardiac myocytes from normal rats. Eur. J. Pharmacol. 767, 1–9. 10.1016/j.ejphar.2015.09.022 26407652

[B41] Sandner P. Zimmer D. P. Milne G. T. Follmann M. Hobbs A. Stasch J. P. (2021). Soluble guanylate cyclase stimulators and activators. Handb. Exp. Pharmacol. 264, 355–394. 10.1007/164_2018_197 30689085

[B42] Schermuly R. T. Stasch J. P. Pullamsetti S. S. MiddendoRff R. Muller D. Schluter K. D. (2008). Expression and function of soluble guanylate cyclase in pulmonary arterial hypertension. Eur. Respir. J. 32 (4), 881–891. 10.1183/09031936.00114407 18550612

[B43] Schmidt H. H. Schmidt P. M. Stasch J. P. (2009). NO- and haem-independent soluble guanylate cyclase activators. Handb. Exp. Pharmacol. 191, 309–339. 10.1007/978-3-540-68964-5_14 19089335

[B44] Schmidt H. H. Walter U. (1994). NO at work. Cell. 78 (6), 919–925. 10.1016/0092-8674(94)90267-4 7923361

[B45] Schorr A. E. (1975). Lotka's law and map librarianship. J. Am. Soc. Inf. Sci. 26 (3), 189–190. 10.1002/asi.4630260308

[B46] Shea C. M. Price G. M. Liu G. Sarno R. Buys E. S. Currie M. G. (2020). Soluble guanylate cyclase stimulator praliciguat attenuates inflammation, fibrosis, and end-organ damage in the Dahl model of cardiorenal failure. Am. J. Physiol. Ren. Physiol. 318 (1), F148–F159. 10.1152/ajprenal.00247.2019 31608671

[B47] Stasch J. P. Evgenov O. V. (2013). Soluble guanylate cyclase stimulators in pulmonary hypertension. Handb. Exp. Pharmacol. 218, 279–313. 10.1007/978-3-642-38664-0_12 24092345

[B48] Stasch J. P. Hobbs A. J. (2009). NO-independent, haem-dependent soluble guanylate cyclase stimulators. Handb. Exp. Pharmacol. 191, 277–308. 10.1007/978-3-540-68964-5_13 19089334

[B49] Stasch J. P. Pacher P. Evgenov O. V. (2011). Soluble guanylate cyclase as an emerging therapeutic target in cardiopulmonary disease. Circulation 123 (20), 2263–2273. 10.1161/CIRCULATIONAHA.110.981738 21606405 PMC3103045

[B50] Stasch J. P. Schmidt P. M. Nedvetsky P. I. Nedvetskaya T. Y. H S A. K. Meurer S. (2006). Targeting the heme-oxidized nitric oxide receptor for selective vasodilatation of diseased blood vessels. J. Clin. Invest. 116 (9), 2552–2561. 10.1172/JCI28371 16955146 PMC1555649

[B51] Tobin J. V. Zimmer D. P. Shea C. Germano P. Bernier S. G. Liu G. (2018). Pharmacological characterization of IW-1973, a novel soluble guanylate cyclase stimulator with extensive tissue distribution, antihypertensive, anti-inflammatory, and antifibrotic effects in preclinical models of disease. J. Pharmacol. Exp. Ther. 365 (3), 664–675. 10.1124/jpet.117.247429 29643251

[B52] Udelson J. E. Lewis G. D. Shah S. J. Zile M. R. Redfield M. M. Burnett J. Jr (2020). Effect of praliciguat on peak rate of oxygen consumption in patients with heart failure with preserved ejection fraction: The CAPACITY HFpEF randomized clinical trial. JAMA 324 (15), 1522–1531. 10.1001/jama.2020.16641 33079154 PMC7576408

[B53] Ullah W. Mukhtar M. Al-Mukhtar A. Saeed R. Boigon M. Haas D. (2020). Safety and efficacy of soluble guanylate cyclase stimulators in patients with heart failure: A systematic review and meta-analysis. World J. Cardiol. 12 (10), 501–512. 10.4330/wjc.v12.i10.501 33173569 PMC7596421

[B54] van Eck N. J. Waltman L. (2010). Software survey: VOSviewer, a computer program for bibliometric mapping. Scientometrics 84 (2), 523–538. 10.1007/s11192-009-0146-3 20585380 PMC2883932

[B55] Wardle A. J. Seager M. J. Wardle R. Tulloh R. M. R. Gibbs J. S. R. (2016). Guanylate cyclase stimulators for pulmonary hypertension. Cochrane Database Syst. Rev. 8, Cd011205. 10.1002/14651858.CD011205.pub2 PMC8502073 27482837

[B56] Wilck N. Markó L. Balogh A. Kraker K. Herse F. Bartolomaeus H. (2018). Nitric oxide-sensitive guanylyl cyclase stimulation improves experimental heart failure with preserved ejection fraction. JCI Insight 3 (4), 96006. 10.1172/jci.insight.96006 29467337 PMC5916255

[B57] Yang Q. Zhai X. Lv Y. (2022). A bibliometric analysis of triptolide and the recent advances in treating non-small cell lung cancer. Front. Pharmacol. 13, 878726. 10.3389/fphar.2022.878726 35721205 PMC9198653

[B58] Zabbarova I. V. Ikeda Y. Kozlowski M. G. Tyagi P. Birder L. A. Chakrabarty B. (2022). Benign prostatic hyperplasia/obstruction ameliorated using a soluble guanylate cyclase activator. J. Pathol. 256 (4), 442–454. 10.1002/path.5859 34936088 PMC8930559

[B59] Zheng X. Zheng W. Xiong B. Huang J. (2018). The efficacy and safety of soluble guanylate cyclase stimulators in patients with heart failure: A systematic review and meta-analysis. Med. Baltim. 97 (41), e12709. 10.1097/MD.0000000000012709 PMC6203591 30313068

